# Effects of Gallic Acid on Fermentation Parameters, Protein Fraction, and Bacterial Community of Whole Plant Soybean Silage

**DOI:** 10.3389/fmicb.2021.662966

**Published:** 2021-05-17

**Authors:** Cheng Wang, Mingyang Zheng, Shuo Wu, Xuan Zou, Xiaoyang Chen, Liangfa Ge, Qing Zhang

**Affiliations:** ^1^College of Forestry and Landscape Architecture, Guangdong Province Research Center of Woody Forage Engineering Technology, Guangdong Research and Development Center of Modern Agriculture (Woody Forage) Industrial Technology, South China Agricultural University, Guangzhou, China; ^2^Guangdong Key Laboratory for Innovative Development and Utilization of Forest Plant Germplasm, State Key Laboratory for Conservation and Utilization of Subtropical Agro-Bioresources, South China Agricultural University, Guangzhou, China; ^3^College of Forestry and Landscape Architecture, South China Agricultural University, Guangzhou, China

**Keywords:** whole plant soybean, fermentation quality, protein fraction, bacterial community, gallic acid

## Abstract

Whole plant soybean (WPS) is a kind of legume resource with characteristics of high nutrition, large biomass, and wide distribution. In the present study, we have investigated the feasibility and effects of gallic acid (GA) on WPS silage quality, nitrogen distribution, tannin content, and bacterial community. The 0.5 and 1% (fresh matter basis) GA were added into WPS for dynamic ensiling (days 3, 7, 14, and 30, respectively). The results showed that the WPS silage with GA addition significantly decreased pH value (6.16–5.38 at ensiling day 30), coliform bacteria count and butyric acid (65.3–62.0 g/kg dry matter at ensiling day 30), and amino nitrogen contents (259–88.2 g/kg total nitrogen at ensiling day 30) and promoted lactic acid (9.62–31.5 g/kg dry matter at ensiling day 30), acetic acid (24.1–85.6 g/kg dry matter at ensiling day 30), and tannin (total phenol and hydrolyzable tannin) contents. Additionally, the GA addition also contributed to the change of bacterial community, where *Firmicutes* and *Lactobacillus* were most abundant on phylum and genus levels, respectively. The above results suggested that GA additive applied in WPS silage was an effective strategy to protect nutrition and improve fermentation quality, and the 1% GA addition showed a better effect.

## Introduction

Soybean (*Glycine max* L. Merrill), one of the most valuable oilseed crops all over the world (Dubey et al., 2018), and its by-product soymeal are unarguably considered the most economical plant proteins, thus are commonly used in animal diets. Whole plant soybean (WPS), including stems, leaves, and fruits, is also nutritious and easy to collect and operate during maturity period. However, WPS is mainly directly used as an inefficient animal feed or a rural fuel or is returned to the field or even arbitrarily discarded in recent years (Liu et al., 2015). Thus, optimizing modulation technology is a crucial step for rational utilization of WPS resources. Ensiling is commonly used to store forage biomass, with the characteristics of easy operation, low cost, and less nutrition loss (Liu et al., 2011). But as the legume, WPS might be difficult to achieve a satisfactory silage quality. That is mainly because of the low water-soluble carbohydrates (WSCs) and insufficient epiphytic lactic acid bacteria (LAB) count of fresh WPS, as WSC contents need higher than 60 g/kg dry matter and epiphytic LAB count not below 5 log_10_ CFU/g fresh matter (FM) for obtaining well-preserved silage (Yang et al., 2016). Otherwise, the silage pH cannot decline rapidly, and undesirable microorganisms like *Clostridia* and *Enterobacter* lead to butyric acid production and proteolysis during ensiling (Silva et al., 2016). Therefore, it is needed to quest some strategies to decelerate even prevent these adverse effects for the purpose of protecting forage nitrogen and improving WPS silage quality (Anuraga et al., 2018).

Gallic acid (GA), with the chemical structure of three phenolic hydroxyl groups and one carboxyl group, is also called 3,4,5-trihydroxybenzoic acid or gallo-tannin (Bharti et al., 2015). Generally, GA exhibits a broad range of antibacterial capability mainly owing to destroy the structural integrity of bacteria (Diaz-Gomez et al., 2013) or inhibit the formation of bacterial biofilm *in vitro* (Kang et al., 2008), and thus it has been widely applied in pharmaceutical and food industries (He et al., 2019a). Meanwhile, GA also shows a positive effect on antioxidant, anti-inflammatory, and anti-tumor (Verma et al., 2013; Hans et al., 2016). As Anuraga et al. (2018) reported the polyhydroxy structure of GA contributes to well protein binding ability whereby reducing proteolysis during ensiling process. Additionally, a certain amount of GA added into the ruminant diet can reduce the emission of methane and nitrous oxide in the rumen, thus improving the efficiency of ruminant feed energy utilization and also reducing greenhouse gas emissions for ecological environment protection. GA also has benefit on animals’ growth performance and health in the feeding practice. Samuel et al. (2017) reported that addition of GA at 75–100 mg/kg into broiler chicks’ diet improved feed conversion efficiency in both the grower and overall periods. And adding 0.1 g/L GA in drinking water can regulate the diversity of microflora of broilers, increase the abundance of *Firmicutes*, and decrease the abundance of *Bacteroidetes*, and thus promote the growth of broilers (Serajus et al., 2017). To sum up, GA might be a candidate of ideal silage additives. However, a few studies were available about the effects of GA additive on WPS silage, especially for dynamic ensiling.

Therefore, WPS was ensiled with and without 0.5 and 1% GA for a fermentation period of 3, 7, 14, and 30 days, and then fermentation quality, nitrogen fraction, and bacterial communities of the WPS silage were evaluated.

## Materials and Methods

### Silage Preparation

Whole plant soybean was planted without herbicides or fertilizers and harvested with an approximately homogeneous size. During the fruiting period, WPS was hand-collected from QILIN experimental field at the South China Agricultural University (23°19′ N, 113°34′ E) in Guangzhou City, Guangdong Province, China. The samples were then immediately chopped into 2–3-cm lengths by the manual cutter (Model 9ZP-3.6, Kaiyue Machinery Company, China). After homogenization, the triplicate samples of raw material were used for determining the chemical composition and microbial populations. And the ensiling treatments were conducted with or without 1 and 2% GA (CAS: 149-91-7, purity ≥ 99%; Shanghai-Macklin, China mainland) on an FM basis. Following mixing WPS and GA thoroughly, about 110 g of silage materials were then packed into polyethylene bags and sealed immediately using a vacuum sealer (Lvye DZ280, Yijian Packaging Machinery Co. Ltd., China). A total of 36 silage bags (4 ensiling stage × 3 treatments × 3 replicates) were kept at room temperature (25–30°C) and randomly sampled on days 3, 7, 14, and 30 of ensiling for analysis of fermentation parameters, protein fractions, and bacterial communities.

### Fermentation Characteristics and Chemical Component Analysis

The methods used in this part were similar to our previous study (Wang et al., 2020).

Hence, 20 g of individual silage sample were homogenized with distilled water (180 ml) in an orbital shaker at room temperature, and the supernatants were then serially diluted from 10^–1^ to 10^–6^. Yeast and mold counts were incubated and counted using Rose Bengal agar at 28°C for 72–120 h. Lactic acid bacteria (LAB) and coliform bacteria were cultured respectively on de Man, Rogosa, Sharpe (MRS) agar and Violet Red Bile agar at 30°C for 48 h. Another 20 g silage sample were mixed with 180ml distilled water and incubated overnight at 4°C, then one aliquot of the filtrate was used to measure pH value with pH meter (PHS-3C, INESA Scientific Instrument Co., Ltd., Shanghai, China).

According to Bai et al. (2020), organic acids including lactic acid (LA), acetic acid (AA), propionic acid (PA), and butyric acid (BA) were also determined by filtrate. The ammonia-N (NH_3_-N) content was detected by the phenol-hypochlorite colorimetric method (Ke et al., 2017). The surplus silages were oven-dried for calculating dry matter (DM) and ground for chemical analysis. And those chemical compounds were analyzed in triplicate and expressed on DM basis. Crude protein (CP) and true protein (TP) were analyzed by Kjeldahl nitrogen analyzer (Kjeltec 2300 Auto Analyzer, FOSS Analytical AB, Hoganas, Sweden) according to the methods of the Association of Official Analytical Chemists (AOAC, 1990). The neutral detergent fiber (NDF) and acid detergent fiber (ADF) were analyzed according to Van Soest et al. (1991). The content of WSCs was detected by anthrone method (Murphy, 1958). Hydrolyzable tannin (HT) was measured by the Folin–Ciocalteu colorimetric as described by Makkar (2010).

### Bacterial Community Sequencing Analysis

The WPS silages were sampled and extracted the total bacterial DNA with a DNA Kit (Omega Biotek, Norcross, GA, United States) following the attached instructions. And specific conducted steps were similar to Bai et al. (2020). The V3–V4 regions of 16S rDNA were amplified using the primers (341F: CCTACGGGNGGCWGCAG; 806R: GGACTACHVGGGTATCTAAT). Polymerase chain reactions (PCRs) were conducted in a 50-μl mixture [5 μl of 10 × KOD Buffer, 1.5 μl of each primer (5 μM), 1 μl of KOD polymerase, 5 μl of 2.5 mM dNTPs, and 100 ng of template DNA] and the same reaction procedures as reported by He et al. (2019a). After purified and quantified, the purified PCR products were sequenced on an Illumina HiSeq 2500 Sequencing System (Illumina, Inc., San Diego, CA, United States), and the raw sequences were analyzed according to the procedures of Wang et al. (2018). The bioinformatic data were analyzed using the free online platform^1^. The α-diversity was calculated in the Quantitative Insights Into Microbial Ecology (QIIME) (version 1.9.1) bioinformatic pipeline (version 1.8.2)^2^. The β-diversity was analyzed with principal component analysis (PCA). And the relative abundances of different bacterial communities at the phylum and genus levels were also analyzed.

### Statistical Analysis

The effects of GA and ensiling days on the fermentation quality and chemical characteristics of WPS silage were analyzed with IBM SPSS 20.0 for Windows statistical software package. The results were evaluated using two-way analysis of variance (ANOVA), with Duncan’s multiple range tests. Statistical significance was determined at the *P* < 0.05 level. All the figures in this paper were downloaded from Omicsmart online platform and further embellished by the software Adobe Illustrator CS 6.0.

## Results

### Characteristics of Fresh Whole Plant Soybean Prior to Ensiling

The chemical compositions and microbial population of fresh WPS prior to ensiling were listed in Table 1. The DM content of WPS was 285 g/kg FM. And the nutrition indexes including CP, TP, NPN, NDF, and ADF were 172 g/kg DM, 840 g/kg TN, 160 g/kg TN, 462 and 274 g/kg DM, respectively. The WSC content of WPS was 44.7 g/kg DM. Moreover, the epiphytic LAB count of fresh WPS in this trial was 5.37 log_10_ CFU/g FM, and the counts for yeasts, molds, and coliform bacteria were 5.02, 4.49, and 5.54 log_10_ CFU/g FM, respectively. The tannin content including total phenol, SP, and HT were 3.55, 1.44, and 1.61 g/kg DM, respectively.

**TABLE 1:** Chemical composition and microbial population of fresh whole plant soybean prior to ensiling (±SD, *n* = 3).

**Items**	**Whole plant soybean**
Dry matter (g/kg FM)	285 **±** 4.9
Crude protein (g/kg DM)	172 **±** 12.8
True protein (g/kg TN)	840 **±** 25.3
Non-protein nitrogen (g/kg TN)	160 ± 25.3
Neutral detergent fiber (g/kg DM)	462 **±** 32.7
Acid detergent fiber (g/kg DM)	274 **±** 27.2
Water soluble carbohydrate (g/kg DM)	44.7 **±** 3.38
Lactic acid bacteria (Log_10_ CFU/g FM)	5.37 **±** 0.12
Yeasts (Log_10_ CFU/g FM)	5.02 **±** 0.06
Molds (Log_10_ CFU/g FM)	4.49 **±** 0.20
Coliform bacteria (Log_10_ CFU/g FM)	5.54 **±** 0.10
Total phenol (g/kg DM)	3.05 **±** 4.26
Simple phenol (g/kg DM)	1.44 **±** 0.28
Hydrolyzable tannin (g/kg DM)	1.61 **±** 0.30

*FM, fresh matter; DM, dry matter; TN, total N; CFU, colony forming units.*

### Fermentation Quality, Microbial Population, and Chemical Compositions of Dynamic Whole Plant Soybean Silage

The DM content, pH value, organic acids content, and microbial population of WPS dynamically ensiled with or without GA are presented in Table 2. The DM content was significantly improved (*P* < 0.05) with GA addition on ensiling days 3 and 30, also increased (*P* > 0.05) on days 7 and 14. The pH value of GA-treated silages was significantly (*P* < 0.05) decreased in whole ensiling stage. And ensiling days had a highly significant effect (*P* < 0.01) on pH decline. However, all treatments of WPS silage showed relatively high pH value (5.38–6.56). Meanwhile, GA addition significantly improved (*P* < 0.05) the LA and AA contents, while these contents significantly decreased (*P* < 0.05) with prolonged ensiling days (except for 1% GA treatment on AA content). The LAB count decreased significantly (*P* < 0.05) with GA addition, and the addition of GA also significantly decreased (*P* < 0.05) other microbes’ number (yeasts, molds, and coliform bacteria). BA content was significantly decreased (*P* < 0.05) with GA addition but significantly increased (*P* < 0.05) with prolonged ensiling days. Table 3 showed the protein fractions and tannin content of WPS dynamically ensiled with or without GA. In our study, the addition of GA had little effect on the content of TP and NPN. And with the prolonged ensiling days, TP content of WPS silage significantly reduced (*P* < 0.05), while NPN and NH_3_-N contents significantly increased (*P* < 0.05). And the addition of GA decreased the NH_3_-N (*P* < 0.05) content of WPS silage. Furthermore, the tannin content including total phenol, SP, and HT all significantly (*P* < 0.05) increased with addition of GA. But with the prolonged ensiling days, their content all significantly (*P* < 0.05) decreased.

**TABLE 2:** Organic acid contents, pH and microbial population of ensiled whole plant soybean.

**Items**	**Treatments**	**Ensiling days**				**SEM**	**Significant**		
		**3**	**7**	**14**	**30**		**D**	**T**	D*T
^2^Dry matter (g/kg FM)	CK	^1^279^*c*^	276	276	265^*b*^	1.95	NS	**	NS
	0.5%GA	291^*b*^	285	294	288^*a*^				
	1%GA	290^*a*^	290	293	294^*a*^				
pH	CK	6.56^*aB*^	6.51^*aA*^	6.18^*aA*^	6.16^*aB*^	0.06	**	**	NS
	0.5%GA	6.13^*b*^	5.99^*b*^	5.86^*ab*^	5.74^*b*^				
	1%GA	5.61^*cA*^	5.58^*cA*^	5.62^*cA*^	5.38^*cB*^				
Lactic acid (g/kg DM)	CK	18.1^*cA*^	11.7^*cB*^	10.4^*cC*^	9.62^*bC*^	4.8	**	**	**
	0.5%GA	64.3^*bA*^	46.9^*bB*^	24.2^*bC*^	14.4^*bC*^				
	1%GA	85.2^*aA*^	72.1^*aA*^	41.6^*aB*^	31.5^*aB*^				
Acetic acid (g/kg DM)	CK	54.7^*bA*^	43.3^*bA*^	26.9^*bB*^	24.1^*cB*^	4.57	**	**	**
	0.5%GA	57.8^*bB*^	93.1^*aA*^	91.4^*aA*^	64.5^*bB*^				
	1%GA	93.7^*a*^	81.4^*a*^	93.4^*a*^	85.6^*a*^				
Propionic acid (g/kg DM)	CK	ND	ND	ND	ND	—	—	—	—
	0.5%GA	ND	ND	ND	ND				
	1%GA	ND	ND	ND	ND				
Butyric acid (g/kg DM)	CK	32.0^*aC*^	40.4^*aB*^	52.0^*aB*^	65.3^*aA*^	2.96	**	**	*
	0.5%GA	8.04^*bC*^	32.4^*aB*^	36.4^*bB*^	43.3^*bA*^				
	1%GA	ND^*cD*^	20.0^*bC*^	29.7^*bB*^	42.0^*bA*^				
Lactic acid bacteria (Log10 CFU/g FM)	CK	8.72^*aA*^	8.92^*aB*^	8.60^*aAB*^	8.36^*aB*^	0.09	**	**	**
	0.5%GA	7.89^*C*^	8.45^*aA*^	8.53^*aA*^	8.10^*bB*^				
	1%GA	7.07^*cC*^	7.76^*bB*^	8.22^*bA*^	7.79^*cB*^				
Molds (Log_10_ CFU/g FM)	CK	3.93^*aA*^	<2.00^*B*^	<2.00^*B*^	<2.00^*B*^	0.14	**	NS	**
	0.5%GA	3.81aA	<2.00^*B*^	<2.00^*B*^	<2.00^*B*^				
	1%GA	2.97^*bA*^	<2.00^*B*^	<2.00^*B*^	<2.00^*B*^				
Yeasts (Log_10_ CFU/g FM)	CK	3.92^*aA*^	2.75^*B*^	<2.00^*C*^	<2.00^*C*^	0.13	**	Ns	NS
	0.5%GA	3.83^*aA*^	2.93^*A*^	2.98^*A*^	<2.00^*B*^				
	1%GA	3.20^*bA*^	2.61^*B*^	2.42^*B*^	<2.00^*C*^				
Coliform bacteria (Log_10_ CFU/g FM)	CK	6.49^*aB*^	7.95^*aA*^	7.79^*A*^	6.36^*aB*^	0.17	**	**	NS
	0.5%GA	6.57^*aB*^	7.62^*bA*^	7.29^*A*^	5.91^*aC*^				
	1%GA	5.45^*bB*^	7.35^*cA*^	6.99^*A*^	4.86^*bB*^				

^1^Means in the same row (^*A–C*^) or column (^*a–c*^) followed by different letters differ (*P* < 0.05). ^2^FM, fresh matter; DM, dry matter; CFU, colony forming units; ND, not detected; “—”, default; SEM, standard error of means; CK, control; 0.5%GA, 0.5% FM gallic acid added; 1%GA, 1% FM gallic acid added; D, ensiling days effect; T, treatments effect; D*T, the interaction effect of treatments and ensiling days. *P < 0.05; **P < 0.01; NS, no significant effect.

**TABLE 3:** Protein fractions and tannin content of ensiled whole plant soybean.

**Items**	**Treatments**	**Ensiling days**				**SEM**	**Significant**		
		**3**	**7**	**14**	**30**		***D***	***T***	D*T
^2^Crude protein (g/kg DM)	CK	167	168	174	169	1.28	NS	NS	NS
	0.5%GA	163	169	178	176				
	1%GA	163	170	162	172				
True protein (g/kg TN)	CK	^1^567^*A*^	488^*B*^	458^*C*^	384^*D*^	12.1	**	NS	NS
	0.5%GA	554^*A*^	486^*B*^	465^*B*^	385^*C*^				
	1%GA	568^*A*^	550^*A*^	445^*B*^	386^*B*^				
Non-protein-N (g/kg TN)	CK	433^*D*^	512^*C*^	542^*B*^	616^*A*^	12.1	**	NS	NS
	0.5%GA	446^*C*^	514^*B*^	535^*B*^	615^*A*^				
	1%GA	432^*B*^	450^*B*^	555^*A*^	614^*A*^				
Ammonia-N (g/kg TN)	CK	38.1^*aC*^	87.9^*aC*^	125^*aB*^	259^*aA*^	11.6	**	**	**
	0.5%GA	16.0^*bC*^	38.2^*bC*^	67.5^*bB*^	112^*bA*^				
	1%GA	10.4^*bC*^	22.9^*bC*^	50.8^*bB*^	88.2^*bA*^				
Total phenol (g/kg DM)	CK	6.05^*bA*^	4.98^*bB*^	3.65^*cB*^	2.50^*bC*^	0.83	**	**	NS
	0.5%GA	14.0^*aA*^	6.88^*bB*^	9.20^*bB*^	5.75^*abB*^				
	1%GA	18.0^*aA*^	12.7^*aB*^	11.5^*aB*^	10.2^*aB*^				
Simple phenol (g/kg DM)	CK	4.17^*aA*^	3.23^*AB*^	2.22^*B*^	2.08^*cB*^	0.31	**	**	**
	0.5%GA	5.11^*aA*^	3.21^*B*^	3.14^*B*^	3.57^*bB*^				
	1%GA	8.27^*bA*^	2.75^*B*^	4.08^*B*^	6.77^*aA*^				
Hydrolyzable tannin (g/kg DM)	CK	1.88^*a*^	1.75b	1.43b	0.43	0.64	**	**	NS
	0.5%GA	8.87^*bA*^	3.67^*bB*^	6.07^*aAB*^	2.19^*B*^				
	1%GA	9.71^*bA*^	9.93^*aA*^	7.40^*aAB*^	3.44^*B*^				

^1^Means in the same row (^*A–D*^) or column (^*a–c*^) followed by different letters differ (*P* < 0.05). ^2^DM, dry matter; TN, total N; SEM, standard error of means; CK, control; 0.5%GA, 0.5% FM gallic acid added; 1%GA, 1% FM gallic acid added; D, ensiling days effect; T, treatments effect; D*T, the interaction effect of treatments and ensiling days. **P < 0.01. NS, no significant effect.

### Bacterial Diversity of Whole Plant Soybean Silage

**FIGURE 1:**
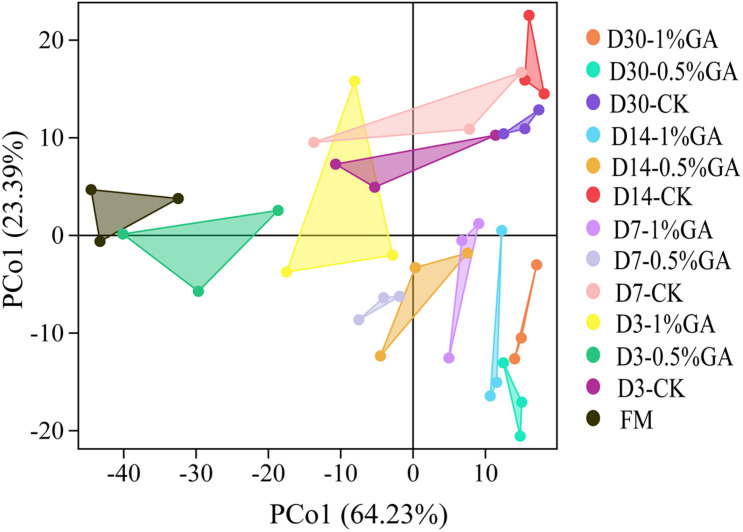
Principal component analysis (PCA) of bacterial communities for whole plant soybean silage with and without the addition of gallic acid (GA) (FM, fresh matter; CK, blank control; 0.5% GA, 0.5% FM GA addition; 1% GA, 1% FM GA addition).

In this study, α-diversity of bacterial community of raw material and dynamically ensiled WPS was shown in Table 4. The good coverage value for all treatments were all above 0.99. And the addition of GA increased the Shannon, Simpson, Chaos, and Ace index compared to contrast check (CK). And the operational taxonomic units (OTUs), richness, and diversity of bacterial communities were significantly increased (*P* < 0.05) with prolonged ensiling days. The result of unweighted principal coordinate analysis (PCoA) was shown in Figure 1. PCoA 1 and PCoA 2 of WPS silage were 64.23 and 24.39%, respectively. Moreover, the bacterial community of WPS ensiled alone showed a clear separation from the samples treated with GA addition.

**TABLE 4:** Alpha diversity of bacterial community of ensiled whole plant soybean.

**Items**	**^2^FM**	**Treatments**	**Ensiling days**				**SEM**	**Significant**		
			**3**	**7**	**14**	**30**		**D**	**T**	D*T
Sobs	884	CK	^1^984^*a*^	938	927	1020^*b*^	25.3	**	*	**
		0.5%GA	900^*bB*^	977^*B*^	914^*B*^	1437^*aA*^				
		1%GA	968a	954	952	1178^*b*^				
Shannon	3.21	CK	4.83	4.80	4.89	5.39^*b*^	0.13	**	NS	NS
		0.5%GA	3.99^*C*^	5.28^*B*^	5.17^*B*^	6.07^*aA*^				
		1%GA	4.66^*B*^	5.36^*AB*^	5.40^*AB*^	6.00^*aA*^				
Simpson	0.66	CK	0.89	0.89	0.87	0.92	0.01	**	*	**
		0.5%GA	0.76^*B*^	0.93^*A*^	0.93^*A*^	0.95^*A*^				
		1%GA	0.88^*B*^	0.94^*A*^	0.94^*A*^	0.96^*A*^				
Chao	1409	CK	1572	1528	1527C	1627^*b*^	28.8	**	*	*
		0.5%GA	1536	1747	1462	2016^*aA*^				
		1%GA	1658	1595	1597	1753^*b*^				
Ace	1415	CK	1593	1553	1598	1684^*b*^	33.2	**	*	*
		0.5%GA	1579^*B*^	1756^*B*^	1525^*B*^	2140^*aA*^				
		1%GA	1720	1619	1637	1898^*ab*^				
Goods-coverage	1.00	CK	0.99	1.00	1.00	0.99	—	NS	NS	NS
		0.5%GA	1.00	0.99	0.99	0.99				
		1%GA	0.99	0.99	0.99	0.99				

^1^Means in the same row (^*A–C*^) or column (^*a–c*^) followed by different letters differ (*P* < 0.05). ^2^FM, fresh soybean straw; CK, control; 0.5%GA, 0.5% FM gallic acid added; 1%GA, 1% FM gallic acid added; SEM, standard error of means; “—”, default; D, ensiling days effect; T, treatments effect; D*T, the interaction effect of treatments and ensiling days. **P < 0.01. NS, no significant effect.

### Bacterial Abundance of Whole Plant Soybean Silage

**FIGURE 2:**
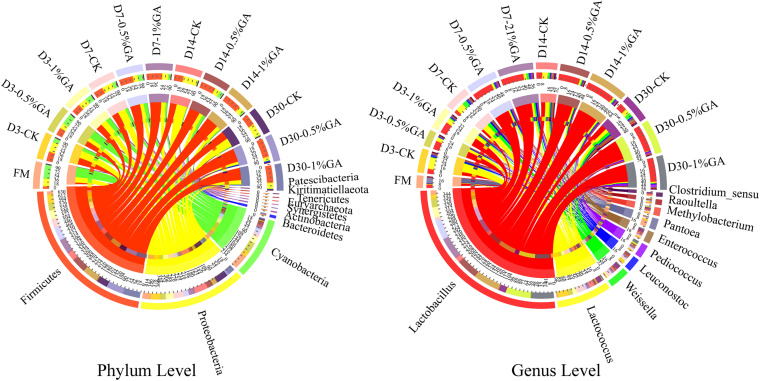
Circos map of bacterial communities at the phylum and genus levels for whole plant soybean silage with and without the addition of gallic acid (GA) (FM, fresh matter; CK, blank control; 0.5% GA, 0.5% FM GA addition; 1% GA, 1% FM GA addition).

**FIGURE 3:**
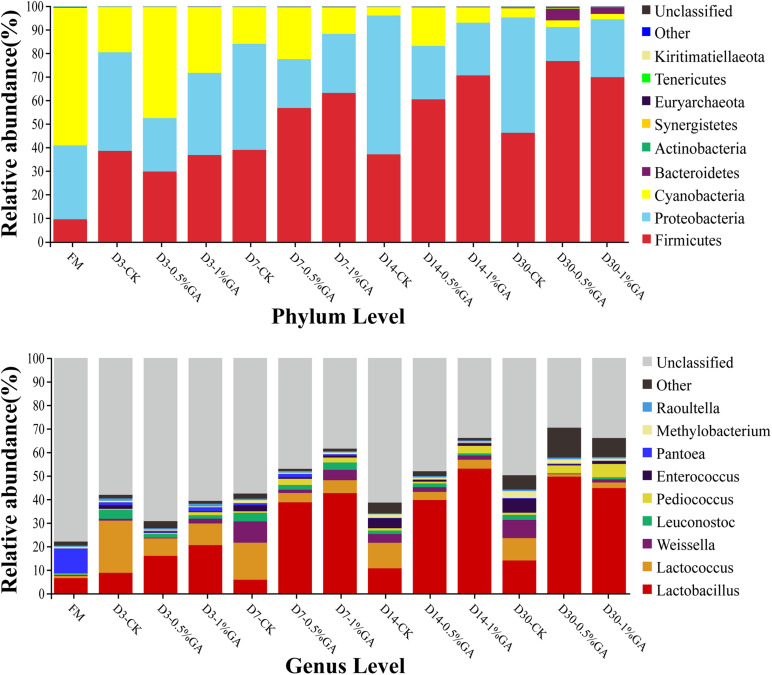
Relative abundance of bacterial communities at the phylum and genus levels for whole plant soybean silage with and without the addition of gallic acid (GA) (FM, fresh matter; CK, blank control; 0.5% GA, 0.5% FM GA addition; 1% GA, 1% FM GA addition).

The relative abundances of bacterial communities of fresh material and WPS silages at the phylum and genus levels were presented in Figure 2 (Circos map) and Figure 3 (accumulation map). As seen in Circos map, *Cyanobacteria* (58.50%), *Proteobacteria* (31.44%), and *Firmicutes* (9.54%) were the top three phyla in the fresh WPS material, while *Firmicutes* (52.25%), *Proteobacteria* (31.92%), and *Cyanobacteria* (14.95%) were also top three dominant phyla in the whole WPS silages. And *Pantoea* (10.57%), *Lactobacillus* (6.46%), and *Lactococcus* (0.95%) were the most dominant genera in the fresh WPS material, but *Lactobacillus* (28.65%), *Lactococcus* (7.92%), and *Weissella* (2.88%) were the most dominant genera in all WPS silages. Figure 3 (accumulation map) shows the abundance and variation of bacterial community in phylum and genus levels. *Cyanobacteria* was the most abundant phylum in fresh material on phylum level. The relative abundance of *Cyanobacteria* decreased, while *Firmicutes* and *Proteobacteria* increased rapidly and became the dominant phyla with prolonged ensiling days. And the relative abundance of *Cyanobacteria* and *Proteobacteria* decreased while *Firmicutes* increased with GA addition. Moreover, *Pantoea* and *Lactobacillus* were the most dominant genera in fresh WPS. However, the relative abundance of *Pantoea* decreased (below 2% in all treatments) during the ensiling process. The most dominant genus in WPS silage was *Lactobacillus*, followed by *Lactococcus*, *Weissella*, *Leuconostoc*, *Pediococcus*, and *Enterococcus*. And the GA additive increased the abundance of *Lactobacillus*, while it decreased the abundance of *Lactococcus* and *Weissella*.

## Discussion

### Characteristics of Fresh Whole Plant Soybean Prior to Ensiling

The DM content of WPS was approximate to ideal DM (30–35%) for satisfactory silage (Guyader et al., 2018). As McDonald et al. (1991) and Muck (2010) reported, high moisture content of fresh material is considered to increase fermentation of undesirable microorganisms, mainly for *Clostridium*, which would result in the nutrition loss of effluent and spoilage during the ensiling process. In general, the WPS is nutrition abundant with high protein content and appropriate fiber content. Especially for high proportion of TP, it means that more valuable protein fraction will be higher efficient in utilization for livestock compared to NPN (He et al., 2019b). However, the protein and fiber contents of WPS were discrepant with those shown by Khorvash et al. (2010). Such variations of the chemical composition of the forage might contribute to the plant varieties, geographical location, climate, fertilization, and season of harvest (Zhang et al., 2016). The sufficient WSC content and epiphytic microbial community (uppermost for LAB) of raw material are the two conclusive factors for well silage quality. The WSC is the fermentation substrate, which is mainly utilized by bacteria to produce organic acid and thus decrease silage pH value. In our study, the WSC content of WPS was not abundant and less than 60–70 g/kg DM, the theoretical requirement for obtaining well-preserved silage (Smith, 1962). Moreover, the epiphytic LAB count of fresh WPS was comparative to 5 log_10_ CFU/g FM, which is necessary to produce well-quality silage (Cai et al., 1998). However, the counts for undesirable fungi and coliform bacteria were relatively high, which indicated that LAB might be difficult to occupy a dominant position at the early stage of ensiling. Furthermore, the HT in WPS might be helpful for undesirable microorganism inhibition (Peng et al., 2017) and extensive proteolysis limitation (Anuraga et al., 2018). To sum up, the WPS was hard to ensile directly because of the insufficient WSC content and high undesirable microbial communities if no silage additives.

### Fermentation Quality, Microbial Population, and Chemical Compositions of Dynamic Whole Plant Soybean Silage

The DM content was improved with GA addition. It indicates GA had a positive effect on DM preservation. As Ávila et al. (2014) reported, the silage DM was mainly consumed by respiration of plant cell and aerobic activity, such as the metabolism of *Clostridia* or yeasts at the early stage of ensiling. He et al. (2019a) also reported dry matter loss of stylo reduced with GA addition after 30 days’ ensiling. In this study, all treatments of WPS silage showed relatively high pH value, which were much higher than 4.2, an indicator of well-preserved silage and thus not suitable for aerobic stability and biomass preservation. Ensiling days decreased the pH value; it was ascribed to the accumulation of organic acid. As well, the pH value of GA-treated silages was decreased throughout the whole ensiling stage. The pH value of silage generally decreases with accumulating of organic acids, which are converted by various microorganisms to ferment WSC. Kung et al. (2018) reported the pH of silages is greatly influenced by acid concentration and the buffering capacity of materials. Therefore, the relative high pH value of WPS silage in our study might be because of the presence alkaline substances like ammonia-N generated from proteolysis or the buffering capacity blocked its decline (He et al., 2019a). Meanwhile, GA addition improved the LA and AA contents. LA is the production of LAB fermentation and charge of pH decline during the early stage of ensiling, while the AA is mainly caused by hetero-fermentative LAB (McDonald et al., 1991). It might be owed to the antibacterial property of GA, which inhibits the undesirable bacteria and benefits LAB fermentation and then reduces negative nutrient competition. Moreover, the content of AA was much higher than LA, especially in the later stage of ensiling. Parvin and Nishino (2009) reported that the growth of AA-producing bacteria might metabolize LA to AA under sugar-deficient conditions. And the AA was an inhibitor of the growth of spoilage microorganisms and increased the aerobic stability (Seglar, 2003). The microbial population presented in Table 2 can further prove the antibacterial effect of GA. The LAB was the dominant microbe in all silages, and its count decreased significantly (*P* < 0.05) with GA addition. This might be caused by decreasing of cocci (such as *Leuconostocs*, *Pediococcus*, *Lactococci*, and *Enterococci*), which was less tolerant to low pH (Cai et al., 1998; Pang et al., 2011). And the addition of GA also decreased yeasts, molds, and coliform bacteria count. By contrast, BA content was decreased with GA addition but increased with prolonged ensiling days. In addition, PA was not detected in all silages. BA is undesirable in silage because of the nutritional damage caused by secondary fermentation as a result of *clostridial* activity (McDonald et al., 1991). In this study, the high BA content might be owed to the abundant coliform bacterial count (4.86–7.95 Log_10_ CFU/g FM), which cannot be restricted in such a high pH condition. According to the above analysis, GA addition is helpful to improve fermentation quality of WPS silage.

The primary purpose of silage-making is to conserve biomass and reduce nutrient loss of fresh forage (Wang et al., 2020). The CP content of WPS silages was comparable to mulberry leaf silage (Wang et al., 2019) but much higher than stylo silage (He et al., 2019a). It indicated that WPS can be utilized as a desirable silage material for the purpose of reducing the waste of biomass resources. In principle, ensiling is a dynamic enzymatic and microbial reaction process, and one of the most important is proteolysis. Proteolysis also called protein degradation, which transforms TP to NPN, mainly some small peptides, amino acid free nitrogen, and NH_3_-N (He et al., 2019a). As a result, proteolysis reduces the silage nutrition and produces terrible smell, especially for high-protein legume. Moreover, excessive rumen-degradable protein cannot be utilized by rumen microorganisms and then discharged by animal excreta (Wang et al., 2019). In the present study, more than half of the protein (840–384 g/kg DM of TP) was degraded before and after ensiling, suggesting that effective strategies should be applied to decelerate or prevent such proteolysis during the ensiling process for the purpose of preserving silage nutrition and protecting the environment by minimizing N emissions (Anuraga et al., 2018). In our study, the addition of GA had litter effect on the content of TP and NPN. The similar results also be reported by He et al. (2019a), the addition of GA decreased TP content while increased NPN content in mulberry leaves and stylo silages. And with the prolonged ensiling days, TP content of WPS silage reduced, while NPN and NH_3_-N content increased. The study of Tao et al. (2012) also reported that proteolysis was the sustained response on the whole ensiling stage. Meanwhile, dipeptidase, carboxypeptidase, and tripeptidyl-peptidase were the principal exopeptidases responsible for silage proteolysis, and their optimal pH values were 8.8, 5.0, and 7.0, respectively. Therefore, the relative high pH value of WPS silage might be responsible for the continuous proteolysis. The NH_3_-N content in silage is a crucial indicator of protein breakdown (Pahlow et al., 2003), which can reflect the silage quality. The addition of GA decreased the NH_3_-N content, it might be due to the potential protein-binding capability of GA. Additional, Mueller (2006) reported tannins had an extensive range of antimicrobial activity. Therefore, GA additive might cause the restriction of deamination of peptides or amino acids in WPS silage and then reduced the generation of NH_3_-N. To sum up, the low proportions of NH_3_-N in the GA-treated group indicate that peptides and free amino acid might account for the abundant NPN. Many studies have proved the bioactivity of peptides and free amino acid on livestock growth and health (Brogden et al., 2003). Thus, the nutrition value of WPS silage was well preserved with GA addition. Surely, the tannin content increased with addition of GA. But with the prolonged ensiling days, their content all decreased. It was the same with He et al. (2019a), who reported that the HT was mostly degraded during mulberry leaf and stylo ensiling. Moreover, the residual GA of WPS silage could be further used to change the N excretion type of cattle and reduce the emission of environmental pollutants, NH_3_ and N_2_O (Getachew et al., 2009). Thus, the positive effect on NH_3_-N restriction indicated that GA additive was effective in preserving protein of WPS silage.

### Bacterial Diversity of Whole Plant Soybean Silage

In order to detect the microbial community’s compositions and abundance (Ni et al., 2019) or monitor the change of microbial community in a dynamically ensiling period (Yang et al., 2018), next-generation sequencing has been widely applied in forage silage. First of all, the good coverage values for all treatments were all above 0.99, indicating that the data from sampling were adequate to represent all of the bacterial communities in the different samples. The bacterial α-diversity of each treatment was estimated by OTUs (Sobs), richness (Chao1 and Ace indexes), and diversity (Shannon and Simpson indexes). In general, the OTUs, richness, and diversity of WPS silage were all higher than fresh WPS. Similar result was also reported by Wang et al. (2019)—higher OTUs, Chaos and Shannon index were exhibited in mulberry leaf silage relative fresh material. It indicated silage fermentation is a dynamic microbe reaction and microbial community structure, diversity, and function will be much variational before and after ensiling (Sepehri and Sarrafzadeh, 2019). Furthermore, the addition of GA increased the Shannon, Simpson, Chaos, and Ace indexes compared to CK. Many studies declared that GA had antibacterial function, while bacterial biodiversity had no change in this study. The reason for this result might be relative high pH value in all silages (>5.32) cannot inhibit the growth of poor adaptability to the acid condition bacteria, such as *Clostridium* (Ni et al., 2017). And the OTUs, richness, and diversity of bacterial community were further increased with prolonged ensiling days. But opposite results have been found by He et al. (2019b), who ensiled *Neolamarckia cadamba* leaves with formic acid and *Lactobacillus farciminis* and indexes of bacterial community (Chaos, Ace, Simpson, and Shannon) in all silages decreased with prolonged ensiling days from 3 to 60. This might ascribe to the relatively low pH value of *Neolamarckia cadamba* leaves silage (<4.31 in all silages), and it further decreased from ensiling days 3 to 60. Therefore, in this study, the GA and ensiling days had no inhibition effect on α-diversity of bacterial community mainly owing to the high pH value of the WPS silage.

The result of unweighted PCoA could reflect the distinction of the bacterial community of each treatment. Especially, a clear separation between GA-treated group and CK group, it suggested that GA had a significant influence on bacterial community. Moreover, a higher extent of separation was formed between fresh material and WPS silages. He et al. (2019a) also reported that GA had an impact on microbial community. Thus, the variations of microbial communities were strongly associated with different silage qualities (Wang et al., 2020). Surely, WPS ensiled with 0.5 and 1% GA showed a better fermentation quality that might be due to the GA-treated group promoting the microbial communities’ composition and abundance and then achieving a better bacterial diversity for desirable silages.

### Bacterial Abundance of Whole Plant Soybean Silage

In the present study, the variational composition of dominant phylum and genus between fresh WPS material and WPS silage in the whole ensiling period indicated that the composition and abundance of bacterial community were much altered before and after ensiling. Same results were also found by Wang et al. (2019), who reported the change of bacterial abundance between raw material and mulberry leaves silage. Accordingly, we have reason to suspect that it might be these specific variations of bacterial community that result in the change of raw material’s chemical composition after the ensiling process.

Therefore, the accumulation map could clearly show the abundance and variation of bacterial community in phylum and genus levels with different treatments. Overall, on the phylum level, *Cyanobacteria* was the most abundant phylum in fresh material. Zhang et al. (2019) also reported similar results. With prolonged ensiling days, the relative abundance of *Cyanobacteria* decreased, while *Firmicutes* and *Proteobacteria* increased rapidly and became the dominant phyla; these even constituted more than 90% of relative abundance on ensiling day 30. Liu et al. (2019) also found that *Firmicutes* and *Proteobacteria* were the most dominant phyla and occupied up to 99% of the total relative abundance at the later period of barley silages. Moreover, the relative abundance of phyla between GA-treated group and CK group was much different and which was dramatically altered by the ensiling days. The relative abundance of *Cyanobacteria* and *Proteobacteria* decreased, while *Firmicutes* increased with GA addition. It was indicated that the bacterial community of WPS silage was remarkably altered by the addition of GA. The study of Heberline (2017) declared that *Cyanobacteria*, a photosynthesizing phylum of bacteria could exist in diverse environments and mainly be researched on the biotechnical and pharmaceutical industries, such as biofuels or fertilizer generation. In this study, with the effect of GA addition and ensiling days, the dominance of *Firmicutes and Proteobacteria* on the phylum level might be because of the relative low pH of WPS silages and anaerobic conditions, which were beneficial to the growth of *Firmicutes* and *Proteobacteria*. The genus-level bacterial communities of WPS silages are also shown in Figure 3. In general, over 75% bacterial community in fresh material was unclassified, whereas more bacterial communities (30.68–70.39%) were identified in WPS silages. It further proved the variation of fermentation parameters was mainly owed to the discrepancy of bacterial communities between fresh material and WPS silages. Moreover, *Pantoea* and *Lactobacillus* were the most dominant genera in fresh WPS. However, the relative abundance of *Pantoea* decreased during the ensiling process. Ogunade et al. (2018) found that *Pantoea* had the ability to reduce NH_3_-N content and pH value in alfalfa silage. But the specific function of *Pantoea* needs to be extensively investigated in various types of forage silage. Furthermore, the most dominant genus in WPS silage was *Lactobacillus*, followed by *Lactococcus*, *Weissella*, *Leuconostoc*, *Pediococcus*, and *Enterococcus*. And the GA additive increased the abundance of *Lactobacillus*, while it decreased the abundance of *Lactococcus* and *Weissella*. Generally, *Lactobacillus*, *Weissella*, *Enterococcus*, and *Lactococcus* are the most commonly used as lactate-producing bacteria in silage and mainly used to occupy the dominant microbe position at the early stage of ensiling and ensure well fermentation quality (Pahlow et al., 2003; Yang et al., 2016; Kuikui et al., 2018). *Lactobacillus* is a rigorous homofermentative LAB and can produce two molecules of LA by decomposing one molecule of glucose. *Lactobacillus* grows rapidly and decreases pH of silage after some plant cell and aerobic microorganisms consume oxygen at the early stage of ensiling; finally, undesirable microorganisms like *Clostridium* are inhibited (Dunière et al., 2013). *Weissella* is thought to be early colonizer microorganisms and be inhibited ultimately by the low pH value (<4.20) due to organic acid accumulation (Graf et al., 2016). Because of the relatively high pH value (>5.38 in all samples) of WPS silage, *Weissella* species was detected in two experiments. Different from us, Wang et al. (2019) detected the bacterial communities of corn stalk silage with the terminal pH of 3.86–3.98 and *Weissella* was not found. Meanwhile, most *Weissella* species are obligate heterofermentative bacteria that mainly convert WSC to lactate and acetate. Thus, the pretty high AA content (>24.1 g/kg DM) in all WPS silages might be due to the abundant *Weissella*. *Enterococcus* is also applied in grass silage as LAB inoculation, it could reduce ammonia N content of silage and directly affect ruminal fermentation by improving ruminal microbial biomass production (Weinberg et al., 2003). The high relative abundance of those genera indicated that acid production accumulated and pH reduced rapidly; therefore, the undesirable microorganisms like *Clostridium* were inhibited and the forage nutrition would be better preserved. Interestingly, *Clostridium* was not detected in our study. *Clostridium* is an undesirable genus during the ensiling process, and many studies have found that it is harmful to forage protein conservation and DM protection and produces satisfactory organic acid and thus prevents rapid fall of silage pH, whereas it promotes the growth of less acid-tolerant spoilage microorganisms (Zheng et al., 2017; He et al., 2019a). Moreover, *Clostridium* is sensitive to pH value. It can grow in conditions of pH over 4.5 and be rapidly inhibited if the pH value falls to 4 or below (Muck, 2010). The similar result found by Wang et al. (2019), *Clostridium* was also not detected in mulberry leaves silage with or without *Lactobacillus casei* and sucrose addition after 30 days’ ensiling. It might be the *Clostridium* in fresh WPS, which was either found in the present study. Surely, in order to explain the occasionally unfound *Clostridium* in silages, a more advanced sequencing technique is required to classify the microbial community at a higher taxonomic level, as 16S DNA full-length sequencing or metagenomic sequencing. In addition, considerable abundance of *Leuconostoc*, *Pediococcus*, and *Methylobacterium* was found in control silage or in the GA-treated silage. Pahlow et al. (2003) reported *Leuconostoc* is also lactate-producing bacteria same as *Lactobacillus* and commonly found in silages. Therefore, the high relative abundance of *Leuconostoc* in groups was beneficial for organic acid accumulation and pH decline and then contributes to more acid-tolerant LAB (mainly for *Lactobacillus*). *Pediococcus* is often found living in association with plant material, dairy products, and foods produced by LAB (Gashe, 1985). And Cai et al. (1998) found that *Pediococcus* was suitable as a potential silage inoculant and more effective than *Lactobacillus casei.* In our study, *Pediococcus* was more abundant in the GA-treated group. The WPS silage with GA addition showed better fermentation quality, which might be explained by the abundance of desirable genera, such as *Pediococcus* and *Lactobacillus*. *Methylobacterium* is strictly neutrophilic and an aerobic bacterium and has also been found in many forage silages (Ogunade et al., 2018; Wang et al., 2019). Holland (1997) declared that *Methylobacterium* is crucial in environmental carbon cycle and contributes to the ability to metabolize plant decomposition compounds. However, the specific function of *Methylobacterium* on silages needs further study.

**FIGURE 4:**
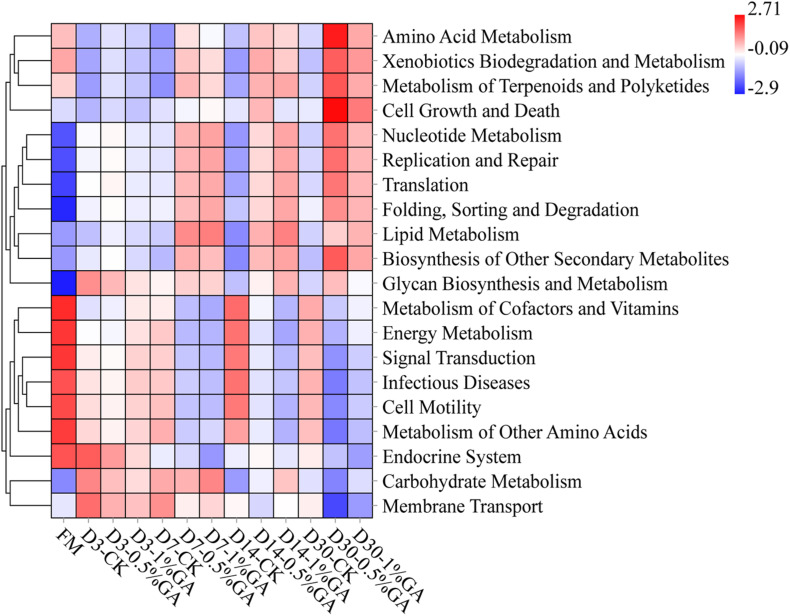
Heatmap of 16S rRNA gene-predicted functional profile for whole plant soybean silage with and without the addition of gallic acid (GA) (FM, fresh matter; CK, blank control; 0.5% GA, 0.5% FM GA addition; 1% GA, 1% FM GA addition).

The 16S rRNA gene-predicted functional heatmap obtained with Tax4Fun on classification level 2 was shown in Figure 4. The discrepancy of bacterial composition and abundance in respective treatment groups might be the key reason for differences of gene-predicted functions. Overall, during ensiling days 7–30, WPS ensiled with the GA addition, the function of amino acid metabolism, xenobiotics biodegradation and metabolism, metabolism of terpenoids and polyketides, nucleotide metabolism, replication and repair translation folding, sorting and degradation, lipid metabolism, biosynthesis of other secondary metabolites all increased, whereas the function of energy metabolism, signal transduction, infectious diseases, cell motility, and metabolism of other amino acids all decreased. It might be the GA additive, which induced the abundant variation of some functional bacteria. For instance, the functions of metabolism of terpenoids and polyketides and the biosynthesis of other secondary metabolites increased; it might be because of the bioactivity of GA. Moreover, the antibacterial characteristic of GA could explain the function of decreased infectious diseases. Above all, GA could be applied in WPS silage to inhibit infectious diseases and improve the biosynthesis of other secondary metabolites for well fermentation quality. But further research is needed to define the other gene-predicted functions.

## Conclusion

This present study showed that pH value, coliform bacteria count, and BA and NH_3_-N contents were decreased, while LA, AA, and tannin (total phenol and HT) contents were increased with addition of GA. Meanwhile, with prolonged ensiling days, the pH value, microbe number, and LA, AA, TP, and tannin contents all decreased, whereas BA, NPN, and NH_3_-N contents increased. The GA also contributes to the change of bacterial diversity, where *Firmicutes* and *Lactobacillus* were most abundant on phylum and genus levels, respectively. The above results suggested that GA additive applied in WPS silage was an effective strategy to protect forage nutrition and improve silage quality, and the 1% GA addition showed a better effect.

## Data Availability Statement

The original contributions presented in the study are included in the article/supplementary material, further inquiries can be directed to the corresponding author/s.

## Author Contributions

CW contributed to the investigation, software, data curation, formal analysis, and writing the original draft. MZ and XZ contributed to the investigation, methodology, visualization, and validation. SW contributed to the investigation and methodology. XZ contributed to the investigation, methodology, visualization, and validation. XC contributed to the conceptualization, funding acquisition, project administration, resources and validation. LG contributed to the materials, investigation, funding acquisition and methodology. QZ contributed to the conceptualization, data curation, project administration, supervision, and validation. All authors contributed to the article and approved the submitted version.

## Conflict of Interest

The authors declare that the research was conducted in the absence of any commercial or financial relationships that could be construed as a potential conflict of interest.

## Footnotes

1 http://www.omicshare.com/tools

2 https://qiime.org
